# Efficacy and Safety of Platelet-Rich Plasma Injections for the Treatment of Female Sexual Dysfunction and Stress Urinary Incontinence: A Systematic Review

**DOI:** 10.3390/biomedicines11112919

**Published:** 2023-10-28

**Authors:** Irina Dankova, Nikolaos Pyrgidis, Maksim Tishukov, Efstratia Georgiadou, Meletios P. Nigdelis, Erich-Franz Solomayer, Julian Marcon, Christian G. Stief, Dimitrios Hatzichristou

**Affiliations:** 1 School of Medicine, Faculty of Health Sciences, Aristotle University of Thessaloniki, 54124 Thessaloniki, Greecetmax@inbox.ru (M.T.); 2 Department of Urology, University Hospital, LMU Munich, 81377 Munich, Germany; julian.marcon@med.uni-muenchen.de (J.M.); christian.stief@med.uni-muenchen.de (C.G.S.); 3 Department of Gynecology & Obstetrics, Buelach Hospital, 8180 Bülach, Switzerland; efstratia.georgiadou@gmail.com; 4 Department of Gynecology, Obstetrics and Reproductive Medicine, Saarland University Hospital, 66421 Homburg, Germany; meletios.nigdelis@uks.eu (M.P.N.); erich.solomayer@uks.eu (E.-F.S.); 5 First Department of Urology, G. Gennimatas Hospital, Aristotle University of Thessaloniki, 54124 Thessaloniki, Greece; d.hatzichristou@gmail.com; 6 Institute for the Study of Urological Diseases, 54622 Thessaloniki, Greece

**Keywords:** female sexual dysfunction, stress urinary incontinence, PRP injections, platelet-rich plasma, systematic review

## Abstract

**Introduction:** There is no clear evidence in the literature that platelet-rich plasma (PRP) injections improve female sexual dysfunction (FSD) and female stress urinary incontinence (SUI). **Objectives:** A systematic review was performed to study the efficacy and safety of PRP injections in women with the above pathologies, as well as to explore the optimal dosing, frequency and area of injections, and duration of treatment. **Methods:** A systematic search on PubMed, Embase and the Cochrane Library database was performed, as well as sources of grey literature from the date of database or source creation to January 2023. After title/abstract and full-text screening, clinical studies on humans evaluating the efficacy of PRP in gynecological disorders using standardized tools were included. Risk of bias was undertaken with RoB-2 for randomized-controlled trials (RCT) and the Newcastle-Ottawa Scale (NOS) for observational studies. **Results:** Four prospective and one retrospective study explored FSD, while six prospective and one RCT evaluated female SUI. A total of 327 women with a mean age of 51 ± 12 years were included. For FSD, PRP significantly improved the Female Sexual Function Index (FSFI), the Vaginal Health Index (VHI) and the Female Sexual Distress score (FSDS). For SUI, PRP led to a significant improvement in the International Consultation on Incontinence Questionnaire—Short Form (ICIQ-SF) and the Urogenital Distress Inventory (UDI-6). The identified RCT reported a significantly higher mean score of ICIQ-SF (*p* < 0.05) and UDI-6 (*p* < 0.01) in the midurethral sling group compared to the PRP injections group. Regarding the risk of bias, the RCT was characterized by high risk, whereas the observational studies were of moderate risk. The protocol for PRP injections for FSD is the injection of 2 mL of PRP into the distal anterior vaginal wall once a month for 3 months. For female SUI, 5–6 mL of PRP should be injected into the periurethral area once a month for 3 months. **Conclusions:** Despite the promising initial results of PRP injections, the level of current evidence is low due to methodological issues in the available studies. It becomes clear that there is an emerging need for high-quality research examining PRP injections for the treatment of FSD and female SUI.

## 1. Introduction

Female sexual dysfunction (FSD) and female stress urinary incontinence (SUI) are two common conditions affecting both women of reproductive age and those over the age of 40 [1,2]. FSD comprises disorders that affect orgasm, sexual interest/arousal and genito-pelvic pain (dyspareunia) [3]. Accordingly, FSD is associated with vulvovaginal atrophy, a condition that causes pain during sexual activity due to estrogen deficiency [4]. Female SUI refers to the involuntary leakage of urine during activities that increase abdominal pressure, such as coughing, sneezing, or exercising. Both conditions can have a significant impact on women’s quality of life and overall well-being [2,5,6].

Platelet-rich plasma (PRP) is a concentrated solution of platelets that is rich in growth factors, showing the ability to facilitate angiogenesis, neuroprotection, neural regeneration, regulation of inflammation and wound healing in preclinical studies, which, in turn, leads to better organ function [7,8]. There are different kits, such as RegenKit^®^ A-PRP, TruPRP^®^ and others, that allow for the preparation of a PRP solution with different platelet concentrations [9]. PRP injection is a relatively recent treatment modality, and therefore, exact data on the dosing of treatment, the location, frequency and duration of its administration are scarce. Nevertheless, the adverse events of PRP therapy, such as infection, bleeding, and nerve damage, appear to be minimal according to the data [10]. Parallel to its evolving role and demonstrated safety in regenerative medicine, the use of PRP is still being explored for the treatment of FSD and female SUI [11]. Based on recent evidence, PRP injections may have the potential to be part of a non-hormonal and surgical treatment approach for patients with FSD due to its regenerative effects by increasing collagen formation and neovascularization in the anterior vaginal wall [12]. Regarding female SUI, preliminary studies suggest that repeated PRP injections into the suburothelium decreased bladder pain and urgency episodes in patients with interstitial cystitis/bladder pain syndrome [13]. Still, despite its favorable properties, data on the role of PRP on the treatment of both pathologies remain limited and heterogenous.

Based on the previous notion, we aimed to summarize, in a holistic approach, the current role of PRP as a treatment modality for FSD and female SUI, and to highlight areas for further research. In this scope, we conducted a systematic review to evaluate the efficacy and safety of PRP injections for improving FSD and SUI, as well as to explore the optimal dosing and duration of treatment, as well as the frequency and area of injections. 

## 2. Materials and Methods

### 2.1. Search Strategy

This systematic review was performed based on the principles of the Cochrane Handbook for Systematic Reviews of Interventions and the PRISMA statement [14,15]. All materials and methods were a priori documented in a protocol registered at PROSPERO (CRD42022384473). Two authors (I.D. and M.T.) systematically searched PubMed, Embase and the Cochrane Library database for studies on humans assessing the role of PRP in FSD and female SUI published from the date of each database creation to January 2023. These researchers also hand-searched important sources of grey literature, including clinical trial registries and published abstracts from major conferences on the matter. They also perused the reference lists of all eligible studies and relevant reviews. Extensive information on the applied search strategy can be found in Supplement Data S1. 

### 2.2. Eligibility Criteria

Our predefined inclusion criteria comprised randomized controlled trials (RCTs) or observational studies assessing symptoms of FSD, or female SUI after PRP injections in adult women using standardized tools on the conditions. Standardized tools considered as validated were the following: Female Genital Self-Image Scale (scores vary from 7 to 28, no cut-off point, higher scores indicate better genital self-image) [16], Female Sexual Distress Scale Revised (scores vary between 0 and 48, a cut-off 15 or higher is an indication of sexual distress) [17], Female Sexual Function Index questionnaire (scores vary from 1 to 36, a score of 26.55 or lower is an indication of sexual dysfunction) [18], Rosenberg Self-Esteem Scale (scores vary from 0 to 30; scores below 15 suggest low self-esteem) [19], Vaginal Health Index (scores vary from 5 to 25, with scores <15 meaning that there is vulvovaginal atrophy) [20], International Consultation on Incontinence Questionnaire-Female Lower Urinary Tract Symptoms (scores vary from 0 to 48, no cut-off point, higher scores indicate more severe the urinary symptoms) [21], International Consultation on Incontinence Questionnaire—Short Form (scores vary from 0 to 21, no cut-off point, higher scores indicate worse urinary symptoms) [22], Urogenital Distress Inventory-6 (scores vary from 0 to 100, no cut-off point, higher scores indicate higher disability) [23], the cough stress test (the results of the test were positive if urine loss occurred while coughing or negative if no urine loss was documented) [24], 1 h pad test (pad weight after 1 h) [25]. On the contrary, studies assessing outcomes in a dichotomous (yes/no) way, studies that included patients receiving PRP after surgery, case reports, animal or cadaveric studies, as well as systematic reviews, meta-analyses, letters to the editor or commentaries were excluded.

### 2.3. Data Acquisition and Risk of Bias

Two authors (I.D. and M.T.) independently implemented a three-step screening of the title, abstract and full text of all identified studies based on the eligibility criteria. Any disagreements were resolved by consensus. Data concerning study and patient characteristics, PRP preparation technique and injected dose, the duration of treatment, as well as outcomes about FSD and female SUI were tabulated in a predefined Microsoft Excel spreadsheet. The risk of bias of all RCTs was evaluated with RoB-2 [26], whereas all other studies were evaluated with the Newcastle–Ottawa Scale (NOS) [27].

### 2.4. Data Synthesis and Statistical Analysis

We undertook a qualitative synthesis of the main data extracted from all included studies. As prespecified, a meta-analysis could not be performed due to the heterogeneity of the included studies in terms of selection criteria and applied treatment modalities. For the purpose of this systematic review, we divided the included studies into two subgroups. In the first group, we included studies assessing the role of PRP on FSD and relevant pathology such as vulvovaginal atrophy, while, in the second group, we included studies assessing the role of PRP on female SUI.

The primary outcome in the first group was the efficacy (the benefit produced by a given treatment under ideal conditions) [28] of PRP on FSD symptoms. Secondary outcomes included the effect of PRP on (i) sexual distress (frustration, anxiety and worry regarding one’s sexual activity) [29]; and (ii) vulvovaginal symptoms (dryness, burning, irritation, the lack of lubrication, discomfort and pain) [30]. Accordingly, the primary outcome in the second group was the efficacy of PRP on female SUI symptoms. Secondary outcomes included the effect of PRP on (i) urge incontinence (sudden compelling urge to void with involuntary leakage of urine) [31]; (ii) urgency (abrupt, strong, often overwhelming and the need to urinate) [32]; and (iii) urine leakage-related quality of life and (iv) bladder function (based on urodynamic measures) [33].

## 3. Results

### 3.1. Study Selection, Study Characteristics and Quality Assessment

**Figure 1:**
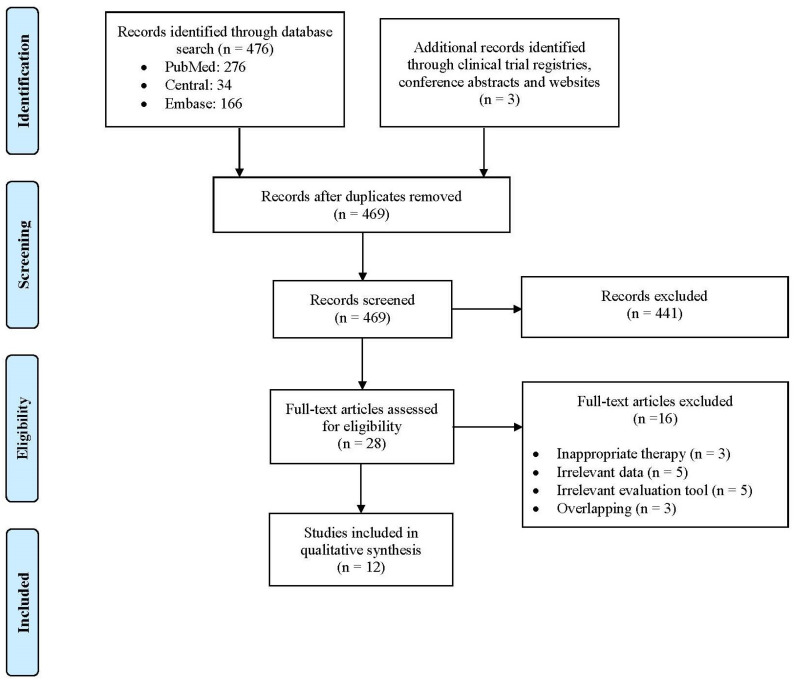
Flow diagram of study selection process. RCT: Randomized controlled trial.

Systematic literature search identified 479 unique studies, a total of 469 records were included after duplicated references had been removed. After title and abstract screening, 28 potentially eligible studies were evaluated as full texts (441 studies were excluded based on our selection criteria). Ultimately, 12 studies (one RCT and 11 observational studies) were included in the qualitative synthesis. Six prospective single-arm studies [34,35,36,37,38,39] and one RCT assessed the efficacy of PRP on female SUI [40], while four prospective single-arm studies [41,42,43,44] and one retrospective single-arm study [12] assessed the efficacy of PRP on FSD. The step-by-step study selection process is illustrated in Figure 1, and the reference list of all excluded studies with reasons for exclusion is presented in Supplement Data S2. Regarding quality assessment, based on RoB-2, the included RCT was considered at high risk of bias (Supplement Data S3) [40]. Accordingly, the ten prospective studies [34,35,36,37,38,39,41,42,43,44] and one retrospective study [12] were considered at moderate risk of bias, based on the NOS (Supplement Data S4).

A total of 327 women with a mean age of 51 ± 12 years were included in the systematic review. Of them, 172 (mean age: 49 ± 11 years) had FSD, and 172 (100%) received PRP therapy, whereas 155 (mean age: 53 ± 13 years) had SUI, and 145 (93.55%) received PRP therapy (Table 1). In the only RCT included in our systematic review, ten patients received periurethral injections of PRP and ten patients received a midurethral sling procedure as per standard treatment for SUI [40]. In all included studies, the follow up after PRP injections was maximum 12 months (Table 1).

### 3.2. PRP Preparation and Application Technique

Across all studies, the PRP injections displayed high heterogeneity in terms of dosage, frequency, and area of administration. All included studies used a standardized approach to prepare PRP, which involved the collection of blood from the participants, the use of anticoagulants, and the centrifugation of blood. Different kits were used in most cases, including the RegenKit^®^ and the TruPRP^®^ (Table 1). In the studies of Apolikhina et al. and Hersant et al., hyaluronic acid was mixed with the PRP to enhance its therapeutic properties [34,44], while in the studies of Amirzagar et al., Runels et al. and Sukgen et al., calcium chloride was mixed with PRP to activate the thrombin cascade and transform the PRP into a platelet-rich fibrin matrix [12,36,41]. The platelet concentration in centrifuged PRP were 1.6 to 6 times higher than the blood levels. 

Regarding the administration protocol, for FSD, the most common injection dose was 2 mL [12,44] and was administered once weekly for 3 months into the distal anterior vaginal wall [12,41]. For female SUI, 5–6 mL of PRP were more frequently injected [34,35,37,38]—once a month for 3 months into the periurethral area [34,35,38,40].

### 3.3. PRP Injections Effect Estimate

In most included studies, PRP injections lead to an improvement of FSD. After treatment with PRP, Sukgen et al. reported a statistically significant improvement in the Female Sexual Function Index (FSFI) from 14 ± 3.8 before treatment to 28 ± 4.8 after treatment (*p* < 0.001). Similarly, Runels et al. reported a mean increase of 5.5 points in the FSFI (*p* = 0.01) after PRP injections [41]. After PRP injections, Hersant et al. demonstrated an improvement from 11 ± 2.1 to 21 ± 4.8 (*p* < 0.001) in the Vaginal Health Index (VHI), and from 36 ± 2.5 to 30 ± 2.5 (*p* < 0.001) in the Female Sexual Distress score (FSDS) after 6 months. Similarly, Saleh et al. showed a significant increase in the total VHI score from 12 ± 2.7 to 17 ± 3.9 (*p* < 0.001) [43].

Female SUI symptoms improved after PRP injections in all seven studies reporting relevant outcomes. After PRP injections, Athanasiou et al. suggested an improvement both in the International Consultation on Incontinence Questionnaire-Female Lower Urinary Tract Symptoms (ICIQ-FLUTS) from 18 ± 9.5 to 12 ± 8.2 (*p* < 0.001) and in the mean urinary leakage at the 1 h pad test from 15 ± 7.9 to 6.2 ± 3.8 g (*p* < 0.001) [35]. Daneshpajooh et al. reported a reduction in the International Consultation on Incontinence Questionnaire—Short Form (ICIQ-SF) from 18 ± 2 to 8 ± 6.8 (*p* < 0.01), in the Urogenital Distress Inventory (UDI-6) from 12 ± 2.5 to 6.6 ± 5.7 (*p* < 0.001), and in the cough stress test from 100% to 30% [40]. Long et al. reported a significant reduction in the ICIQ-SF from 11.5 ± 3 to 7.3 ± 4.3 (*p* < 0.01) and in the UDI-6 from 39 ± 14 to 28 ± 17 (*p* < 0.01) [37]. Similarly, Tahoon et al. confirmed an improvement both in the ICIQ-SF from 12 ± 2.9 to 5 ± 1.4 (*p* < 0.01) and in the UDI-6 from 35 ± 6.9 to 16 ± 4.6 (*p* < 0.01) [39].

Concerning adverse events of PRP injections, only two included studies have provided evidence on the matter. Runels et al. reported the occurrence of extreme sexual arousal (sexual arousal with urination, continuous sexual arousal, ejaculatory orgasm, and spontaneous orgasm) in two patients during treatment with PRP for FSD [41]. Chiang et al. also reported urinary retention that required self-catheterization for several days in one patient after treatment with PRP for SUI [38]. Nevertheless, these adverse events resolved without further treatment.

Regarding the comparison of PRP injections with other treatment methods, Daneshpajooh et al. demonstrated significantly better outcome measures in cases treated with midurethral sling vs. PRP injections. The authors reported a mean ICIQ-SF score of 8 ± 6.8 in the PRP injections group and 2.2 ± 3.5 in the midurethral sling group three months after treatment (*p* = 0.02). Further, the RCT demonstrated that at three months after treatment, the UDI-6 mean score was 6.6 ± 5.7 in the PRP injections group, and 1.3 ± 1.7 in the midurethral sling group (*p* = 0.007) [40]. 

## 4. Discussion

Our systematic review suggests that the use of PRP might improve symptoms of FSD and female SUI. Based on standardized patient-reported outcomes, PRP injections seem to improve sexual function and symptoms of female SUI. Nevertheless, it should be highlighted that, due to the clinical heterogeneity, safe conclusions about the efficacy of PRP cannot be drawn. The most common protocol for PRP injections for FSD is the injection of 2 mL of PRP into the distal anterior vaginal wall once a month for 3 months. On the contrary, for female SUI, 5–6 mL of PRP should be injected into the periurethral area once a month for 3 months.

Importantly, across the included studies, validated questionnaires for FSD, such as the FSFI and the FSDS, were implemented only by two of the seven studies. Accordingly, validated questionnaires for SUI, such as the ICIQ-UI SF, were not used by the majority of the studies, and objective measures, such as the pad test, were rarely reported. The Heterogeneity of reporting methods renders drawing conclusions rather problematic, even though the effectiveness of PRP in FSD and female SUI has been demonstrated. Adding to this, data from studies analyzing other female pelvic diseases (e.g., female pelvic organ prolapse, vesicovaginal fistula and vaginal mesh exposure), erectile function and male SUI suggest that PRP injections may improve symptoms [45,46,47,48,49,50].

There is no consensus in the literature about how many times and how often PRP administrations should be performed [12]. We attempted to determine the ideal dose, frequency, duration, and site of injection of PRP in women with FSD and female SUI. It seems that, for FSD, the distal anterior vaginal wall should be preferred since there is immunohistochemical evidence of greater nerve and microvessel abundancy than the posterior or proximal anterior vaginal wall [51,52]. Thus, PRP injections can contribute to the maximum positive regenerative effect due to neovascularization of the vaginal wall rich in blood vessels, and as a result, increased collagen formation, thereby improving the condition of the vagina [12]. Regarding SUI, five of the six included studies applied periurethral injections, due to the beneficial effect of PRP in terms of regeneration, angiogenesis and neuroprotection on the defective anatomical components of the urethra [53]. Nevertheless, it should be highlighted that further evidence is needed to confirm the validity of the proposed injection areas.

PRP injections have been associated with no serious adverse events, such as infection, bleeding, or nerve damage in multiple studies on wound care, orthopedics, urology, dental surgery and cosmetic procedures [12,54]. The latter is in line with our systematic review, which found that none of the included patients who received PRP injections developed serious adverse events and less than 1% of all patients (3/317) developed minor adverse events.

There is currently variation in the clinical management of FSD and female SUI. Guidelines suggest that FSD and female SUI should only be corrected by physiotherapy or surgery [55,56]. PRP injections are not included in the current guideline recommendations due to the lack of RCTs [55,56]. Within this framework, our findings suggest that PRP injections are safe and may be implemented, at investigational setting, as an additional treatment modality for FSD and female SUI, especially in cases where surgery is not wished for, and physiotherapy does not suffice.

It should be stressed that our findings were mitigated by multiple limitations. First of all, the included studies displayed significant heterogeneity in terms of PRP preparation technique, dose, injected area, and duration of treatment and, therefore, a meta-analysis could not be performed. Of interest, most included studies raised methodological concerns. This problem predominantly stemmed from the small number of included participants, the suboptimal methods of reporting randomization, the relatively short follow-up, the restricted number of events and the implementation of non-recognized scales. Accordingly, some important parameters, such as PRP preparation technique, dose of PRP, injected area, as well as percentage of patients with improvement in underlying disease symptoms, remained unreported in some of the included studies.

## 5. Conclusions

Our findings indicate that PRP injections may be considered for clinical research on FSD. Additionally, in patients with female SUI, PRP injections may contribute to an improvement in the insufficiency of the urethral closure apparatus. Still, the level of evidence for all outcomes was deemed low due to the methodological concerns raised in most of the included studies. Therefore, only the optimal dosage, frequency, the duration of treatment and the injected area of PRP can be inferred. High-quality RCTs should be conducted for the administration of PRP injections to become a high-quality evidence alternative.

## Figures and Tables

**Table 1:** Baseline and estimate characteristics of the included studies.

Study	Type of Study	Disorder	Participants/Participants Receiving PRP (n)	PRP Preparation Technique	Injected Area	PRP Injection Technique	Effect Estimate	Follow-Up
Amirzargar et al., 2016 [36]	Prospective single-arm	SUI	30/30	n/a	Vagina	12 mL; once	Incontinence score based on questionnaire—17 ± 1.8 → 5.9 ± 2.5	No
Apolikhina et al., 2018 [34]	Prospective single-arm	SUI	19/19	RegenKit^®^	Urethra	6 mL. 12 patients—2 times in 6 months; 6 patients—once; 1 patient—3 times in 9 months every 3 months	The cough stress test was negative in 18 patients	12 months
Athanasiou et al., 2021 [35]	Prospective single-arm	SUI	20/20	RegenKit^®^	Anterior vaginal wall, periurethral area	5.5 mL; twice in 4–6 weeks	ICIQ-FLUTS—18 ± 9.5 → 12 ± 8.2; 1 h pad test—15 ± 7.9 g → 6.2 ± 3.8 g	No
Chiang et al., 2022 [38]	Prospective single-arm	SUI	26/26	n/a	Urethral sphincter	5 mL; 4 times in 3 months monthly	UDI-6—5.1 ± 2.3 → 3.2 ± 2.5	12 months
Daneshpajooh et al., 2021 [40]	RCT (comparator: midurethral sling procedure)	SUI	20/10	n/a	Urethra	3 mL. 7 patients—once; 2 patients—twice in 1 month; 1 patient—3 times in 2 months monthly	ICIQ-SF—18 ± 1.9 → 8.0 ± 6.8; UDI-6—12 ± 2.5 → 6.6 ± 5.7. The cough stress test—Positive: 100%, → Positive: 30%	3 months
Hersant et al., 2018 [44]	Prospective single-arm	FSD	20/20	RegenKit^®^	Posterior wall of the vagina, posterior wall of the introitus	2 mL, once	VHI—11 ± 2.1 → 19 ± 3.8; FSDS-R—36 ± 2.5 → 30 ± 2.5	6 months
Long et al., 2021 [37]	Prospective single-arm	SUI	20/20	RegenKit^®^	Anterior vaginal mucosa	5 mL; 4 times in 3 months monthly	ICIQ-SF—12 ± 3 → 7.3 ± 4.3; UDI-6—39 ± 14 → 28 ± 17	6 months
Romashchenko et al., 2022 [42]	Prospective single-arm	FSD	52/52	n/a	Paraurethral zone, introitus vagina, vagina	6 mL; twice in 21–22 days	Vsmax—1.6–2.3 cm/sec at rest and 3.1–4.1 cm/sec in 30 min after video-erotic stimulation → 3.7–5.8 cm/sec at rest and 5.3–8.5 cm/sex after the stimulation	No
Runels et al., 2014 [41]	Prospective single-arm	FSD	11/11	RegenKit^®^, TruPRP^®^	Anterior vaginal wall, clitoris	5 mL once	FSFI—24 → 30; FSDS-R—17 → 7.3	No
Saleh et al., 2022 [43]	Prospective single-arm	FSD	37/37	RegenKit^®^	Posterior vaginal wall, vulva (labia majora, labia minora and vestibular fossa)	4 and 8 mL, twice in 1 month	VHI—12 ± 2.7 → 17 ± 3.9	1 month
Sukgen et al., 2019 [12]	Retrospective single-arm	FSD	52/52	n/a	Anterior vaginal wall, clitoris, paraurethral vaginal wall.	2 mL, 5 times every 4 weeks for 4 months	FSFI—14 ± 3.8 → 28 ± 4.8; FGSIS—17 ± 5.6 → 24 ± 2.2; FSDS-R—19 ± 12 → 11 ± 1.9; RSES—21 ± 6.1 → 22 ± 5.7	6 months
Tahoon et al., 2022 [39]	Prospective single-arm	SUI	20/20	n/a	Anterior vaginal wall, paraurethral vaginal wall	4 mL once	ICIQ-SF—12 ± 2.9 → 5 ± 1.4; UDI-6—35 ± 6.9 → 16 ± 4.6	3 months

FGSIS: Female Genital Self-Image Scale; FSD: female sexual dysfunction; FSDS-R: Female Sexual Distress Scale Revised; FSFI: Female Sexual Function Index questionnaire; ICIQ-FLUTS: International Consultation on Incontinence Questionnaire-Female Lower Urinary Tract Symptoms; ICIQ-SF: International Consultation on Incontinence Questionnaire—Short Form; IR: index resistance; RSES: Rosenberg Self-Esteem Scale; SUI: stress urinary incontinence; UDI-6: Urogenital Distress Inventory-6; VHI: Vaginal Health Index; Vsmax: Maximum speed of blood flow in clitoris vessels.

## Supplementary Materials

The following supporting information can be downloaded at: https://www.mdpi.com/article/10.3390/biomedicines11112919/s1. Supplement Data S1: PubMed search syntax and search string; Supplement Data S2: Reference list of all excluded studies with reasons for exclusion; Supplement Data S3: Risk of bias of RCTs; Supplement Data S4: Risk of bias in non-RCTs. References [13,47,57,58,59,60,61,62,63,64,65,66,67,68,69,70] are cited in the supplementary materials.
