# Supplementary material for: Efficacy and Safety of Platelet-Rich Plasma Injections for the Treatment of Female Sexual Dysfunction and Stress Urinary Incontinence: A Systematic Review
Source: Biomedicines. 2023 Oct 28;11(11):2919. doi: 10.3390/biomedicines11112919 (PMC10669888; doi:10.3390/biomedicines11112919)
Supplement: Supplementary file 1 [file biomedicines-11-02919-s001.zip › biomedicines-2661785-supplementary.pdf]

Data Supplement

Table of Contents

Data Supplement 1: PubMed search syntax and search string 2

Data Supplement 2: Reference list of all excluded studies with reasons for exclusion.........4

Data Supplement 3: Risk of bias of RCTs...5

Data Supplement 4: Risk of bias in non-RCTs..6

Data Supplement 1: PubMed search syntax and search string

Search syntax

ID	Search
#1	pharmacol res perspect [Journal]
#2	prp [All Fields]
#3	platelet rich plasma [MeSH Terms]
#4	platelet rich [All Fields]
#5	plasma [All Fields]
#6	AND #4-5
#7	platelet [All Fields]
#8	rich [All Fields]
#9	plasma [All Fields]
#10	AND #7-9
#11	platelet rich plasma [All Fields]
#12	OR #1-3,6,10-11
#13	orgasm [MeSH Terms]
#14	orgasm [All Fields]
#15	orgasmic [All Fields]
#16	orgasms [All Fields]
#17	vagina [MeSH Terms]
#18	vagina [All Fields]
#19	vaginas [All Fields]
#20	vagina s [All Fields]
#21	vaginae [All Fields]
#22	incontinance [All Fields]
#23	incontinence [All Fields]
#24	incontinences [All Fields]
#25	incontinency [All Fields]
#26	incontinent [All Fields]
#27	incontinents [All Fields]
#28	clitoris [MeSH Terms]

#29 clitoris [All Fields]

#30 rejuvenate [All Fields]

#31 rejuvenated [All Fields]

#32 rejuvenates [All Fields]

#33 rejuvenating [All Fields]

#34 rejuvenation [MeSH Terms]

#35 rejuvenation [All Fields]

#36 rejuvenations [All Fields]

#37 rejuvenative [All Fields]

#38 rejuvenator [All Fields]

#39 rejuvenators [All Fields]

#40 OR #13-39

#41 #12 AND #40

Search string

("pharmacol res perspect"[Journal] OR "prp"[All Fields] OR ("platelet rich plasma"[MeSH Terms] OR ("platelet rich"[All Fields] AND "plasma"[All Fields]) OR "platelet rich plasma"[All Fields] OR ("platelet"[All Fields] AND "rich"[All Fields] AND "plasma"[All Fields]) OR "platelet rich plasma"[All Fields])) AND ("orgasm"[MeSH Terms] OR "orgasm"[All Fields] OR "orgasmic"[All Fields] OR "orgasms"[All Fields] OR ("vagina"[MeSH Terms] OR "vagina"[All Fields] OR "vaginas"[All Fields] OR "vagina s"[All Fields] OR "vaginae"[All Fields]) OR ("incontinance"[All Fields] OR "incontinence"[All Fields] OR "incontinences"[All Fields] OR "incontinency"[All Fields] OR "incontinent"[All Fields] OR "incontinents"[All Fields]) OR ("clitoris"[MeSH Terms] OR "clitoris"[All Fields]) OR ("rejuvenate"[All Fields] OR "rejuvenated"[All Fields] OR "rejuvenates"[All Fields] OR "rejuvenating"[All Fields] OR "rejuvenation"[MeSH Terms] OR "rejuvenation"[All Fields] OR "rejuvenations"[All Fields] OR "rejuvenative"[All Fields] OR "rejuvenator"[All Fields] OR "rejuvenators"[All Fields]))

The search strategy was modified accordingly for the other databases.

Data Supplement 2: Reference list of all excluded studies with reasons for exclusion

Duplicate publications or data presented in part and then in whole in future publications

[1] Apolikhina I, Gorbunova E, Saidova A, Sukhikh G. Prp injections in the treatment for stress urinary incontinence. International Urogynecology Journal, 2017 Jan;28(1):S233-S234.

[2] Kalantzis C, Athanasiou S, Zacharakis D, Petrakis V, Pontikaki A, Grigoriadis T. The role of platelet rich plasma (PRP) for treatment of stress urinary incontinence. Female Pelvic Medicine and Reconstructive Surgery, 2021 Jan;27(10):E664-E664.

[3] Manin E, Taraschi G, Berndt S, Martinez de Tejada B, Abdulcadir J. Autologous Platelet-Rich Plasma for Clitoral Reconstruction: A Case Study. Arch Sex Behav. 2022 Jan;51(1):673-678. doi: 10.1007/s10508-021-02172-9. Epub 2021 Nov 15. PMID: 34779981; PMCID: PMC8858308.

Inappropriate therapy

[1] Al-Hamadani IT, Shehab E mahmod, aziz NA. Comparative Changes in Sexual Dysfunction of Married Women after Colpoperineorrhaphy Versus Colpoperineorrhaphy with Additional Platelet Rich Plasma Injection. Indian Jour of Foren Med & Toxicol 2019;13:314. https://doi.org/10.5958/0973-9130.2019.00309.8.

[2] Luksenburg A, Barcia JJ, Sergio R, Fernandez S, Pelosi MA, Pelosi MA. Stress Urinary Incontinence: Treatment With Platelet-Rich-Plasma Injection and Placement of Polydioxanone Threads—A Pilot Study. Am J Cosmet Surg 2022;39:76–84. https://doi.org/10.1177/0748806821998100.

[3] Medel S, Alarab M, Kufaishi H, Drutz H, Shynlova O. Attachment of Primary Vaginal Fibroblasts to Absorbable and Nonabsorbable Implant Materials Coated With Platelet-Rich Plasma: Potential Application in Pelvic Organ Prolapse Surgery. Female Pelvic Med Reconstr Surg. 2015 Jul-Aug;21(4):190-7. doi: 10.1097/SPV.0000000000000178. PMID: 25900058.

Irrelevant data

[1] Aguilar P, Hersant B, SidAhmed-Mezi M, Bosc R, Vidal L, Meningaud JP. Novel technique of vulvo-vaginal rejuvenation by lipofilling and injection of combined platelet-rich-plasma and hyaluronic acid: a case-report. SpringerPlus 2016;5:1184. https://doi.org/10.1186/s40064-016-2840-y.

[2] Jiang Y, Lee P, Lee Y, Kuo H. Urethral sphincter injections of platelet-rich plasma (PRP) in treatment of urinary incontinence due to intrinsic sphincteric deficiency refractory to conventional treatment. Neurourology and Urodynamics, 2019 Jan;38(0):S262-S263.

[3] Jiang YH, Lee PJ, Kuo HC. Therapeutic Efficacy of Urethral Sphincter Injections of Platelet-Rich Plasma for the Treatment of Stress Urinary Incontinence due to Intrinsic Sphincter Deficiency: A Proof-of-Concept Clinical Trial. Int Neurourol J. 2021 Mar;25(1):51-58. doi: 10.5213/inj.2040272.136. Epub 2021 Jan 19. PMID: 33504126; PMCID: PMC8022168.

[4] Khera B. Efficacy of PRP in genitourinary syndrome of menopause: A prospective study. BJOG: An International Journal of Obstetrics and Gynaecology, 2021 Jan;128(0):214-215.

[5] Willison FB, Nguyen T. Innovative treatment of uterovaginal prolapse with autologous membrane from platelet rich plasma. BJOG: An International Journal of Obstetrics and Gynaecology, 2021 Jan;126(0):235-235.

Irrelevant evaluation tool

[1] Behnia-Willison F, Pour NR, Mohamadi B, Willison N, Rock M, Holten IW, O'Shea R, Miller J. Use of Platelet-rich Plasma for Vulvovaginal Autoimmune Conditions Like Lichen Sclerosus. Plast Reconstr Surg Glob Open. 2016 Nov 23;4(11):e1124. doi: 10.1097/GOX.0000000000001124. PMID: 27975027; PMCID: PMC5142493.

[2] Castellani D, Valloni A, Piccirilli A, Paradiso Galatioto G, Vicentini C. An innovative approach to treating vaginal mesh exposure after abdominal sacral colpopexy: endoscopic resection of mesh and platelet-rich plasma; initial experience in three women. Int Urogynecol J. 2017 Feb;28(2):325-327. doi: 10.1007/s00192-016-3154-x. Epub 2016 Sep 21. PMID: 27655187.

[3] Goldstein AT, King M, Runels C, Gloth M, Pfau R. Intradermal injection of autologous platelet-rich plasma for the treatment of vulvar lichen sclerosus. J Am Acad Dermatol. 2017 Jan;76(1):158-160. doi: 10.1016/j.jaad.2016.07.037. PMID: 27986140.

[4] Kim SH, Park ES, Kim TH. Rejuvenation Using Platelet-rich Plasma and Lipofilling for Vaginal Atrophy and Lichen Sclerosus. J Menopausal Med. 2017 Apr;23(1):63-68. doi: 10.6118/jmm.2017.23.1.63. Epub 2017 Apr 28. PMID: 28523261; PMCID: PMC5432469.

[5] Posey LK, Runels C. In office surgery and use of platelet rich plasma for treatment of vulvar lichen sclerosus to alleviate painful sexual intercourse. Journal of Lower Genital Tract Disease, 2017 Jan;21(4):S14-S14.

Data Supplement 3: Risk of bias of RCTs

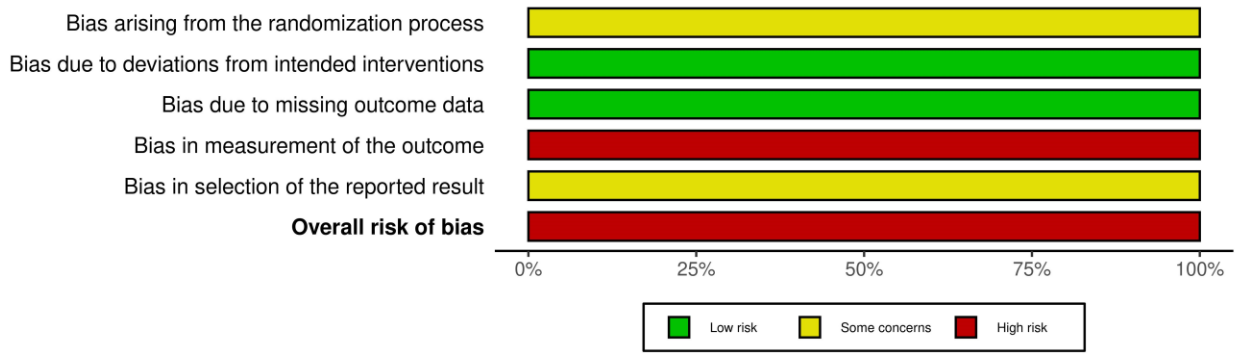

Data Supplement 3.1: Overall risk of bias assessment according to the RoB-2 for RCTs.

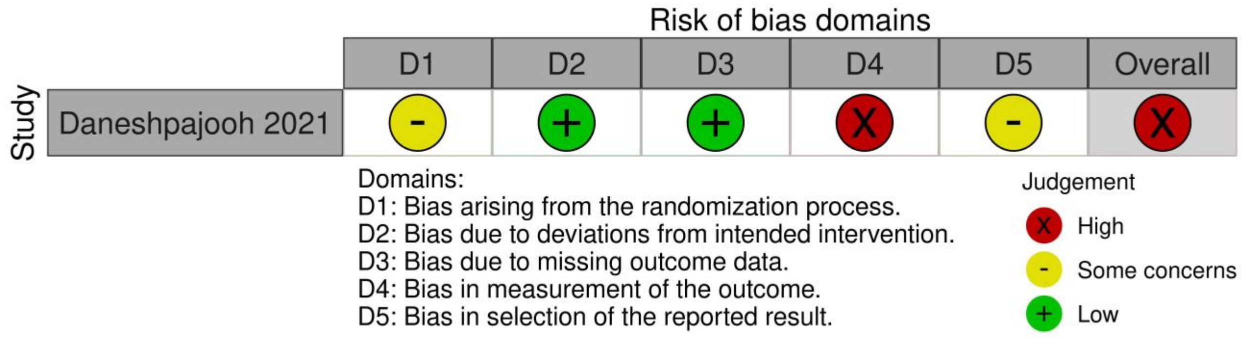

Data Supplement 3.2: Study-by-study risk of bias assessment according to the RoB-2 for RCTs.

Data Supplement 4: Risk of bias in non-RCTs

Study	Selection				Comparability	Outcome			Total selection score	Total comparability score	Total outcome score	Quality
	**Representativeness of the exposed cohort**	**Selection of the non exposed cohort**	**Ascertainment of exposure**	**Demonstration that outcome of interest was not present at start of study**	**Comparability of cohorts based on design or analysis**	**Assessment of outcome**	**Follow-up long enough for outcome to occur**	**Adequacy of follow up of cohorts**				
Amirzargar 2016	1			1	2		1	1	2	2	2	Moderate
Apolikhina 2018	1			1	2		1	1	2	2	2	Moderate
Athanasiou 2021				1	2		1	1	1	2	2	Moderate
Chiang 2022	1			1	2		1	1	2	2	2	Moderate
Hersant 2018	1			1	2		1	1	2	2	2	Moderate
Long 2021	1		1	1	1		1	1	3	1	2	Moderate
Romashchenko 2022				1	2		1	1	1	2	2	Moderate
Runels 2014	1			1	2		1	1	2	2	2	Moderate

Study	Selection				Comparability	Outcome			Total selection score	Total comparability score	Total outcome score	Quality
	Representativeness of the exposed cohort	Selection of the non exposed cohort	Ascertainment of exposure	Demonstration that outcome of interest was not present at start of study	Comparability of cohorts based on design or analysis	Assessment of outcome	Follow-up long enough for outcome to occur	Adequacy of follow up of cohorts				
Saleh 2022	1		1	1	2		1		3	2	1	Moderate
Sukgen 2019	1			1	2		1	1	2	2	2	Moderate
Tahoon 2022	1			1	2		1	1	2	2	2	Moderate

Data Supplement 4: Study-by-study risk of bias assessment according to the Newcastle-Ottawa Scale (NOS) in included non-RCTs.
